# Interleaved practice benefits implicit sequence learning and transfer

**DOI:** 10.3758/s13421-021-01168-z

**Published:** 2021-04-01

**Authors:** Julia M. Schorn, Barbara J. Knowlton

**Affiliations:** Department of Psychology, University of California, Los Angeles, Los Angeles, CA 90095 USA

**Keywords:** Contextual interference, Serial reaction time task, Implicit learning

## Abstract

Compared to blocked practice, interleaved practice of different tasks leads to superior long-term retention despite poorer initial acquisition performance. This phenomenon, the contextual interference effect, is well documented in various domains but it is not yet clear if it persists in the absence of explicit knowledge in terms of fine motor sequence learning. Additionally, while there is some evidence that interleaved practice leads to improved transfer of learning to similar actions, transfer of implicit motor sequence learning has not been explored. The present studies used a serial reaction time task where participants practiced three different eight-item sequences that were either interleaved or blocked on Day 1 (training) and Day 2 (testing). In Experiment 1, the retention of the three training sequences was tested on Day 2 and in Experiment 2, three novel sequences were performed on Day 2 to measure transfer. We assessed whether subjects were aware of the sequences to determine whether the benefit of interleaved practice extends to implicitly learned sequences. Even for participants who reported no awareness of the sequences, interleaving led to a benefit for both retention and transfer compared to participants who practiced blocked sequences. Those who trained with blocked sequences were left unprepared for interleaved sequences at test, while those who trained with interleaved sequences were unaffected by testing condition, revealing that learning resulting from blocked practice may be less flexible and more vulnerable to testing conditions. These results indicate that the benefit of interleaved practice extends to implicit motor sequence learning and transfer.

## Introduction

From the moment we wake up to the moment we fall asleep, we perform many skills that we have learned over time, like brushing teeth, typing, driving a car, or playing a musical instrument. Complex and simple skills alike rely on motor dexterity, sequence learning, perceptual acuity, and both explicit and implicit learning (Shmuelof & Krakauer, 2014). Procedural skill learning is a persistent and crucial part of the human experience, so determining an optimal practice schedule is essential as we are heavily dependent on our ability to learn new skills throughout our lifetime. Practice schedules that introduce high contextual interference (CI) by interleaving, or randomizing, tasks hinder initial performance but aid in long-term skill retention. While the CI effect is robust, it has yet to be reliably demonstrated in implicit motor sequence learning and transfer. Determining if the CI effect persists in the absence of explicit knowledge is essential in order to design effective practice schedules, especially for clinical populations who demonstrate impaired explicit memory but preserved implicit memory. For example, patients with amnesia show intact motor sequence learning but impaired declarative memory (Reber & Squire, 1994). Optimizing practice schedules to enhance long-term retention and transfer of learning will benefit lives across a broad spectrum of settings.

Decades of cognitive psychology research have demonstrated that CI through interleaved, or random, practice (compared to blocked practice) is a way to effectively acquire skills that can be retained in the long-term (Battig, 1966; Magill & Hall, 1990; Shea & Morgan, 1979). Interleaving tasks or stimuli can be thought of as a “desirable difficulty” as it hinders initial performance but results in superior long-term retention and transfer performance (Bjork, 1994). Blocked practice, on the other hand, facilitates acquisition performance because it requires the learner repeatedly performs a single task before moving on to the next one. In one of the earliest demonstrations of the benefits of CI in motor skill learning, subjects practiced three sequential arm movement tasks, which were either blocked together or intermixed in an interleaved condition (Shea & Morgan, 1979). While performance was worse in the interleaved condition during practice, subjects showed better retention and transfer to similar tasks. Blocked practice usually facilitates faster acquisition but results in poorer long-term retention. This differential effect exemplifies the difference between learning and performance, a critical distinction (for a review, see Soderstrom & Bjork, 2015). For example, a pianist may quickly master a difficult passage by practicing it repeatedly only to forget all progress the next day, because conditions that enhance performance may not enhance learning.

At first glance, the CI effect seems to be at odds with the Specificity of Learning hypothesis, which posits that learning is most optimal when the practice conditions during the acquitision phase are the same as those during the testing phase (Barnett et al., 1973). Some findings in the motor learning literature suggest there is partial support for this hypothesis, though the CI effect is more robust (Shea & Kohl, 1990; Travlos, 2010). Thus, interleaved practice shows benefits even when tasks are blocked at test.

## Theorized mechanisms of the contextual interference effect

Though the benefits of contextual interference are well documented, the mechanism by which interleaving facilitates this enhanced retention is still not completely understood. Two general hypotheses currently stand out. The forgetting-reconstructive hypothesis (or action plan reconstruction hypothesis) posits that interleaving is beneficial because it requires each task set to be frequently retrieved, while during blocked practice the task set remains in working memory for the entire task duration (Lee & Magill, 1983; Lee et al., 1985). When practicing interleaved motor sequences, a pattern must be learned and then immediately “dumped” from working memory in order to prepare for subsequent trials (Lee & Simon, 2004). In each trial, the learner must retrieve a motor pattern into working memory or construct one from scratch. Conversely, blocked motor sequences can remain in working memory for multiple trials without needing to be updated. While frequently forgetting and retrieving stimuli may initially hinder performance, it also allows the learner to practice reconstructing motor patterns, which is beneficial for long-term retention. This hypothesis may partially explain the dissociation between learning and performance often seen in the CI effect (for a review, see Kantak & Winstein, 2012).

On the other hand, the elaboration-distinctiveness hypothesis (or discriminative-contrast hypothesis) posits that interleaved practice facilitates organizational and item-specific processing, so that subjects frequently compare different stimuli for more durable encoding (Shea et al., 1985; Shea & Zimny, 1983). When tasks or stimuli are interleaved, differences between them may be more easily discerned as compared to when they are blocked.

Though traditionally pitted against one another, these two theories are not mutually exclusive and may explain different components of the CI phenomenon (Lin et al., 2008). In an fMRI study, interleaved practice of a motor sequence task compared to blocked practice resulted in increased activation in sensorimotor regions, followed by a decreased activation in similar regions during a delayed retention test (Lin et al., 2011). The increased activation during encoding of interleaved sequences was interpreted as representing additional motor program reloading. Since there was increased retrieval processing during encoding, retrieval at test required less activation in these regions. This view lends credence to the forgetting-reconstructive hypothesis.

However, it appears that interleaving may also offer greater benefits than simply requiring more retrieval. In a study where participants learned to identify paintings by artist in either a blocked or interleaved fashion to develop a concept of different artists’ styles, participants in the interleaved condition showed better learning than those who studied the paintings in a blocked but spaced schedule (Kang & Pashler, 2012). As temporal spacing of study items required explicit retrieval of the artist’s names, inferior performance in the blocked condition indicated that retrieval practice alone cannot account for the benefits of interleaving. Participants had to abstract information across paintings to learn artists’ styles that could be used to classify new paintings, so the contrasts and comparisons made at encoding in the interleaved condition may have been important for generalization, supporting the elaboration-distinctiveness view.

Neither of these theories specifically account for implicit processes important for motor skill learning, and research on the benefits of interleaving has largely ignored the interplay between implicit and explicit learning (Bjork & Kroll, 2015; Shanks & St. John, 1994). In light of this, an alternate theory has emerged that proposes that high CI and increased task switching overloads an individual’s working memory capacity, preventing them from gaining explicit task-relevant knowledge (Rendell et al., 2011) and enabling greater implicit learning. Previous research showing increased levels of cognitive activity with interleaved practice as compared to blocked practice lends some support to this theory (Li & Wright, 2000). Recent findings extend this idea, suggesting that high cognitive effort seen in interleaved practice may be partially due to increased error processing as well as task-switching (Broadbent et al., 2017). Following an error, greater cognitive activity could be attributed to an individual updating and correcting a rule as well as retrieving information for the upcoming task. However, evidence supporting this hypothesis is sparse and relegated to perceptual-cognitive skills. It is also unclear whether CI simply enables more acquisition of implicit knowledge, or if it leads to implicit knowledge that is better retained and better able to support transfer to similar tasks. In the present study, implicit learning under blocked and interleaved conditions will be compared directly.

## The contextual interference effect and implicit motor sequence learning

Research concerning the CI effect and implicit motor learning has largely focused on gross motor skills like those used when playing sports (French et al., 1990; Goode & Magill, 1986; Menayo et al., 2010). Furthermore, research investigating the CI effect in fine motor sequence learning has almost exclusively focused on explicit memory (Wright et al., 2016). Though previous research has explored the effect of CI in implicit motor learning, few studies specifically investigate fine motor sequence learning over a substantial delay (Dang et al., 2019; Sekiya, 2006) and is thus a primary aim of this paper. One such study examined the CI effect in a pursuit-tracking task (Sekiya, 2006). The experimenters told participants in the explicit group the presentation order and number of patterns in the task, while those in the implicit group received no instruction. Interestingly, they failed to replicate the CI effect and found no differences between implicit and explicit learners. One possibility for this finding is the relatively high CI in the Blocked group may have reduced differences between the practice conditions. Blocked segments were intermixed with random segments so that participants did not become aware of the repetition. Thus, it is not yet clear if implicit and explicit learning are similarly affected by CI during practice.

## Implicit motor sequence transfer

Positive transfer to novel tasks or contexts is a crucial goal in many training situations as one often cannot train on every possible task variation or in every possible context. It is suggested that when positive transfer occurs, a memory representation of the skill has been created that is more general than a representation that could only support the practiced sequence. Positive transfer would indicate that participants learned not only the practiced sequences but also generalized knowledge that benefits the performance of new sequences. Learning may also be sequence-specific, with performance of new sequences similar to performance at the beginning of practice on the original sequences. However, learning could also result in negative transfer with performance of new sequences impaired due to interference (Obayashi, 2004). In this case, learning may be sequence-specific, and this sequence knowledge may impair the ability to perform similar sequences.

Though the benefits of interleaving on retention are well-studied, there is currently less evidence that interleaving can also lead to improved transfer to similar actions (Bangert et al., 2014; Brady, 2004; Meira & Tani, 2001; Russell & Newell, 2007; Schmidt & Lee, 2005). Transfer in the skill learning domain has been extensively studied, including transfer of learning from one effector to another (Kelso & Zanone, 2002), such as right hand to left hand, or scaling, such as performing a skilled action at a different rate or using greater force (Newell, 1996). However, there are limited transfer studies on fine motor skills, and none that specifically consider implicit sequence learning over a long delay. Müssgens and Ullén (2015) showed that interleaved sequences, as compared to blocked, led to better transfer to a new sequence on an immediate test, but it is unlikely that these sequences were implicitly learned as a majority of subjects reported some sequence awareness. Additionally, the immediate test made it likely that interference occurred on the test with the sequences that had just been practiced. Similarly, interleaving may reduce negative transfer of motor sequences when new sequences are performed (Shimizu et al., 2016). However, transfer was tested at the end of the experiment, and not after a one day delay when the effects of contextual interference are most apparent. This delay is also crucial for observing “offline gains” in which motor memory is stabilized and improved in the absence of practice and is influenced by sleep (Nader et al., 2000; Walker et al., 2002). It was also unclear whether sequence knowledge was primarily explicit or implicit in Shimizu et al. (2016).

Other studies concerning the CI effect in novel motor skill learning found that prior experience with interleaved practice improved new motor task acquisition, however, delayed retention was unaffected by practice schedule (Hodges et al., 2014; Kim et al., 2016, 2018). Hodges et al. (2014) found that random practice experience, compared with blocked practice, led to better acquisition of three novel motor skills after a one-day delay and mitigated the low accuracy cost usually associated with random practice, but ultimately did not affect retention. Using a similar paradigm, Kim et al. (2016) found that interleaved practice of a motor skill was beneficial for novel skill acquisition, but afforded no retention benefits after a delay. However, interleaved practice in this experiment was not truly random and was more analogous to a blocked practice schedule, as participants practiced 5–15 repetitions of the same motor sequence in a 30-s trial. Kim et al. (2018) sought to address this limitation by using the discrete sequence production (DSP) task to induce high levels of contextual interference and found that acquisition of a novel task was better for individuals with prior interleaved rather than blocked practice, and this benefit remained after a significant delay. Notably, awareness for the sequences was not probed in any of these experiments and sequence learning was presumed to be explicit. Though participants may not always possess explicit, in-depth knowledge of the task, the DSP task is considered to be an explicit sequence learning paradigm and participants are often informed that they are performing fixed motor sequences (Abrahamse et al., 2013; Bo & Seidler, 2009). In light of this, our second experiment aims to examine if contextual interference can aid in positive transfer of novel motor sequences when learning is implicit. Demonstrating the CI effect in transfer of sequence learning to different (but similar) sequences would show that this effect persists despite a high degree of potential interference, similar to real-life learning.

## The current experiment

The proposed study aimed to add to the body of research on contextual interference by examining the effect of interleaved practice on implicit learning of sequences in a serial reaction time task (SRTT). In this task, participants can acquire knowledge of a sequence of locations as shown by more rapid responses to locations presented in sequence compared to those presented in a random order (Nissen & Bullemer, 1987). Participants can show sequence-specific improvement without conscious awareness that the locations appeared in any sequence. Additionally, participants might show non-sequence-specific learning, in that learning and performance of new sequences may be faster than initial performance. Though the SRTT typically examines learning that is specific to the practiced sequence, we argue that learning that generalizes to new sequences may be more relevant to real-world skill learning. In both experiments, training and testing comprised of blocked and interleaved practice conditions, allowing us to examine transfer-appropriate processing (TAP) effects (Morris et al., 1977). TAP effects are related to the Specificity of Learning hypothesis which states that performance is optimal when the context during testing resembles the context during training (Barnett et al., 1973). There is some support of this hypothesis in the motor learning literature; however, the CI effect is more robust and does not always align with this principle, in that test performance in either condition is often better when preceded by interleaved practice (Shea & Kohl, 1990; Travlos, 2010).

## General method

### Overview

We examined the effect of practice schedule on motor sequence learning and transfer by either blocking or interleaving the presentation of three different eight-item motor sequences in a two-day experiment utilizing the SRTT. Both experiments thus had four groups, based on condition, for Day 1 (Train) and Day 2 (Test): Blocked training/Blocked test (BB), Interleaved training /Interleaved test (II), Blocked training/Interleaved test (BI), and Interleaved training/Blocked test (IB). At the end of the experiment, explicit knowledge of the sequences was probed with a questionnaire.

### Behavioral task

The SRTT has frequently been used to measure implicit learning (Keele et al., 2003; Robertson, 2007). In this simple task, the participants are asked to respond to cued locations using keypresses. The participant is instructed to respond to the successive locations as quickly and accurately as possible and is not informed that there is a structure governing the order of the appearance of the cued locations. Despite the lack of awareness of the structure, reaction time (RT) is faster for sequences that were practiced compared with RT for a random presentation of cued locations. RT is the primary dependent measure because error rates are generally low and accuracy is not a useful measure of motor learning in this task. This task may share many features of real-world skills that engage fine motor circuits in which movement components must be produced in a specific sequence, such as typing or playing a musical instrument.

### Study design

We utilized a between-subjects design with the SRTT. Subjects sat with four fingers of the right hand on four keys on a keyboard (U, I, O, P) that corresponded to the four outlined, unfilled circles on a blue computer screen in a darkened room. One of the circles turned white to act as a cue to press the corresponding key (i.e., the first circle on the screen corresponds with “U”). After the button press, another circle turned white and the first circle reverted to being unfilled. A tone sounded if a subject failed to press a button or if a subject made an incorrect button press. RT and accuracy were recorded for each key press. No baseline performance was measured, since this task is simple and baseline performance does not reliably capture individual differences in learning (Stark-Inbar et al., 2017).

**Fig. 1:**
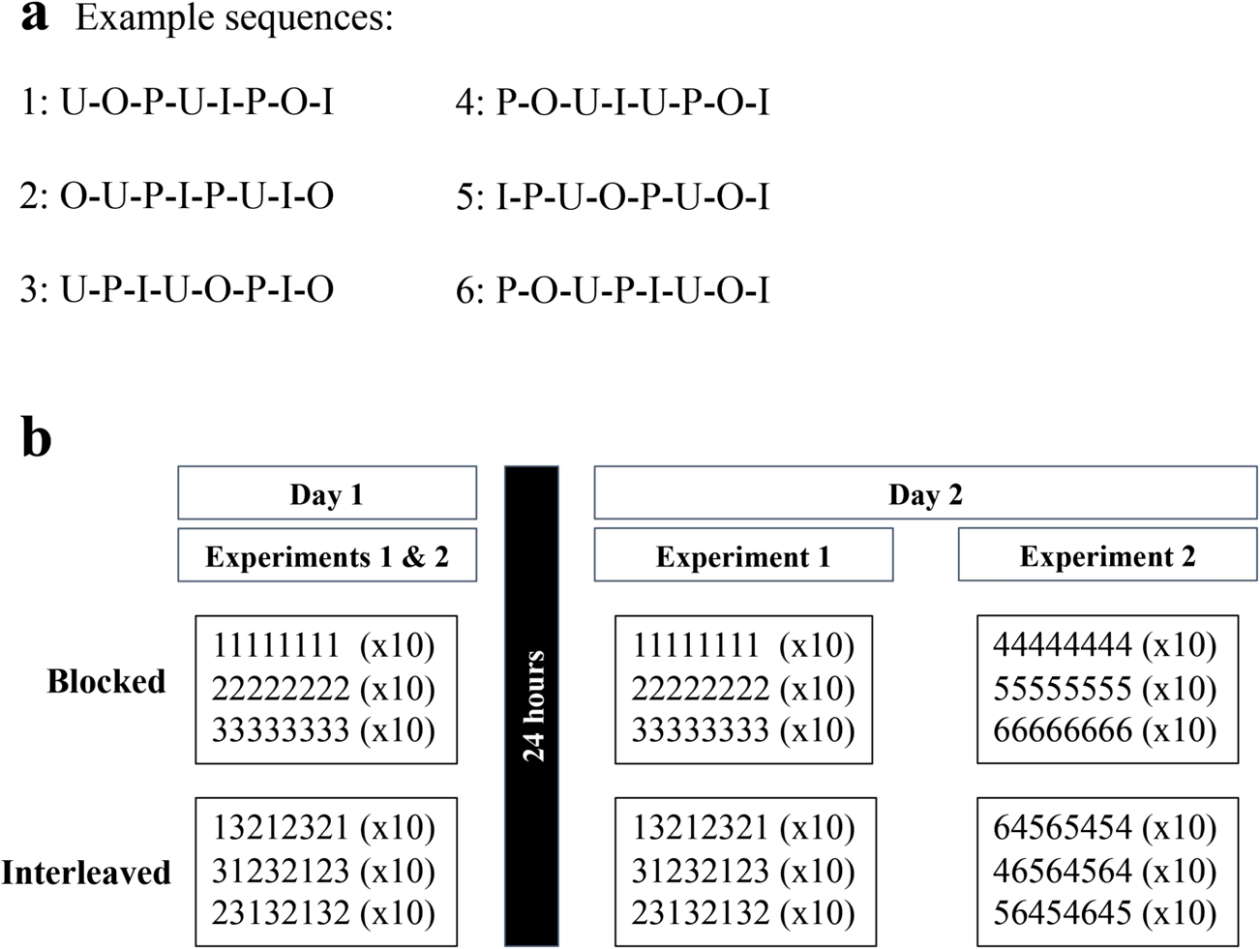
Sequences and study design. (**A**) Six example sequences. In Experiment 1, participants learned three sequences; in Experiment 2, participants learned six sequences – three on Day 1 and three novel sequences on Day 2. (**B**) Study design for both experiments. Day 1 is the same for both while Day 2 is separated out by experiment. Each number represents an eight-item sequence as shown in (**A**)

Participants practiced three different eight-item sequences that were either interleaved or blocked on Day 1 (training) and Day 2 (testing) (Fig. 1). To control for specific item effects, the sequences were randomized so that no two participants had the same ones. Each sequence contained each letter (U, I, O, P) twice. Critically, participants were not told that there were sequences, only to respond to each cue as quickly and as accurately as possible. Participants were randomized into the Interleaved or Blocked training groups and were tested in either a Blocked or an Interleaved condition, counterbalanced across training conditions. Participants were not told which condition they were assigned to on either day. In the blocked condition, participants received 80 repeated presentations of each sequence (i.e., AAA…BBB...CCC). In the interleaved condition, they received three sequences interleaved for a total of 240 trials (i.e., ACBABCBAC....). This number of trials is comparable to the training procedure used in our previous work (Lin et al., 2010).

Day 2 was the same as Day 1, participants were randomly assigned to either the blocked or the interleaved condition. In Experiment 1, the sequences presented on Day 2 to each participant were the same as those presented on Day 1. In Experiment 2, the sequences presented on Day 2 were novel, so participants practiced three sequences on Day 1 and three different sequences on Day 2, for a total of 6 unique sequences. Each day in each experiment contained the same number of trials.

To assess whether subjects had acquired explicit knowledge of the sequences, a free-recall questionnaire based on past research was administered after the second session (Robertson et al., 2004; Willingham & Goedert-Eschmann, 1999). Though free recall may also reflect a degree of implicit memory, that is also true of most explicit awareness tests (Shanks & St. John, 1994). Though there is no consensus on the best way to measure awareness in this task (Robertson, 2007), free recall, rather than recognition, was assessed as it can be argued that it is the most appropriate measure of explicit memory (Frensch & Rünger, 2003). The test was comprised of the three following questions, which prompted the participants to recall the sequences:

1. Did you notice any pattern(s)?
2. How many sequences where there?
3. Please type in all sequences (using the same keys) and hit Enter after each sequence.

Participants were also asked how many hours of sleep they got in between Day 1 and Day 2. Sleep has been shown to be critical for offline gains in implicit learning, however, rapid consolidation of a motor skill can occur within hours or even seconds after practice (Bönstrup et al., 2019; Kim & Wright, 2020; Squire et al., 2015; Walker et al., 2002). We chose a 24-h interval between training and testing to measure long-term retention as this is sufficient to observe consolidation; subsequent nights of sleep (e.g., 48–72 h from learning) seem to maintain the memory and prevent it from decay, with only minimal performance gains, if any at all (Rickard et al., 2008; Walker et al., 2003). There is no consensus on the optimal delay between training and delayed retention testing, but this length of delay is typically used as it is a more practical way to assess consolidation (Kantak & Winstein, 2012). Contextual interference effects are also more apparent after this length of delay (Cahill et al., 2001; Perez et al., 2005).

### Monte Carlo simulation

The implicit-explicit memory distinction may lie on a continuum, with participants having varying amounts of explicit knowledge. However, since we were primarily interested in purely implicit learners, we dichotomized our sample and post hoc sorted participants based on a cutoff determined by a Monte Carlo simulation a priori. This allowed us to determine the number of sequential elements that would be recalled by chance. Subjects would be considered fully implicit learners if their recall was near chance level, and subjects would be considered as having some explicit sequence knowledge if they exceeded this level. This procedure is similar to past research with the SRTT in which a cut-off score based on chance performance was applied to participants’ item recall to define implicit and explicit groups (Robertson et al., 2004; Willingham & Goedert-Eschmann, 1999). Like the present study, this was to ensure that the individuals in the implicit group had little to no awareness of the sequence.

To determine the amount of explicit knowledge for practiced sequences, we created a score for each participant based on their percentage correct recall for each sequence and averaged the three scores, with 1 as a perfect score. For example, if a subject remembered four sequential items of one sequence (0.5), two sequential items of the second sequence (0.25), and none of the third sequence (0), those three scores would be averaged to create a subject’s “explicit score.” In this example, this subject would have a score of 0.25, meaning that on average, they remembered two items per sequence.

Participants were considered to have only implicit knowledge of practiced sequences if their explicit knowledge of the sequences was near chance levels. To determine chance levels in terms of the number of sequential elements produced, we ran a Monte Carlo simulation. We compared three randomly generated “test” sequences to a set 1,000,000 randomly generated sequences. The test sequences and the comparison set of sequences were in the same format as used in the experiment (i.e., eight item sequences, using each character (U, I, O, P) twice). The percentage similarity score for each test sequence was calculated averaging across all items in the comparison set. We ran this simulation 10,000 times. The generated percentage scores all fell within 0.23–0.26. Thus, on average, we concluded that chance level of performance would be producing about two sequential items per sequence (.25). We considered a participant to have minimal knowledge of the sequences if the participant was able to produce three or fewer sequential items per sequence and used this a-priori cut off to dichotomize our sample. Implicit learners were participants who recalled, on average, 0–3 sequential items per sequence (at chance) while explicit learners recalled 4 or more items per sequence (above chance).

## Experiment 1

### Method

#### Participants

100 right-handed young adults were enrolled in the study (88 female; age 18–48 years, *M* = 20.9, *SD* = 4.2). Participants were undergraduate students recruited from UCLA and were given course credit for their participation. All participants gave informed consent using an institutionally approved consent form. Participants were excluded if they had a history of neurological or psychiatric disease or if they were taking neuroactive medication that could affect sensory processing, movement, or cognition. Since the SRTT is a simple task, accuracy likely reflects the degree of participants’ effort, not learning. Thus, participants were excluded if they had an accuracy lower than 80% on either day, in line with previous research (Willingham et al., 2000). All participants reported they they were right-handed, although the degree of handedness was not assessed. A total of 17 participants were excluded for either low accuracy (*n* = 8), computer error (*n* = 5), or failing to complete the experiment (*n* = 4). Our final subject pool consisted of 83 right-handed young adults (59 female, age 18–43 years, *M* = 20.6, *SD* = 3.2).

#### Data analysis

Sequence RTs were calculated by summing the eight key press RTs for correct sequences. In line with previous research, only accurate sequences were analyzed (Reber & Squire, 1998). We took an average of the last ten sequence RTs per sequence (A, B, and C) for the blocked training condition, for a total of 30 trials. For the interleaved training condition, we took an average of sequence RTs from the last 30 trials. For the blocked testing condition, we used the same procedure but looked at the *first* ten trials for each sequence, for a total of 30 trials. Similarly, for the interleaved testing condition, we studied the *first* 30 trials. To measure retention, difference scores were calculated by subtracting the RT of the last 30 trials from Day 1 from the RT of the first 30 trials from Day 2. A negative difference score, reflecting a decrease in RT, indicates improvement (faster performance) from Day 1 to Day 2, while a positive difference score, reflecting an increase in RT, indicates a decline in performance (slower performance) from Day 1 to Day 2. A difference score of zero would indicate successful retention. We assessed learning during the practice phase by looking at sequence RTs over Day 1 (training) in both implicit and explicit learners using the Mann-Kendall test, a nonparametric test for monotonic trends. We also calculated a learning difference score to assess performance at the beginning and end of training. Here, we compared the first 30 trials of interleaved sequences to the last 30 trials, and the first ten of each blocked sequence the last ten of each. Difference scores were calculated similarly to past research (Lin et al., 2010; Wymbs & Grafton, 2009). We conducted one-sample t-tests for all groups (BI, II, BB, IB ) to assess if difference scores were significantly different from zero, which would indicate an improvement or a decline in performance from Day 1 to Day 2.

To examine whether the different practice conditions yielded different levels of sequence awareness, we compared the average number of sequence items that participants recalled using an independent t-test. We conducted a 2 × 2 × 2 between-subjects ANOVA, after post hoc sorting subjects into implicit and explicit learners based on their recall score. Training condition (Interleaved, Blocked), testing condition (Interleaved, Blocked), and learner type (Implicit/Explicit) were the three factors while the difference score was the dependent measure. In addition, we conducted an ANCOVA with recall score as the covariate before sorting participants into implicit or explicit learners. Like the ANOVA, training and testing condition were the two factors and the difference score was the dependent measure.

### Results

On Day 1, a Mann-Whitney test indicated that participants who practiced interleaved sequences were significantly less accurate (*M* = 92.34, *SD* = 4.38) than participants who practiced blocked sequences (*M* = 94.22, *SD* = 4.13; *U* = 1134.50, *p* = .013). However, on Day 2, there was no significant difference in accuracy between those who performed interleaved sequences (*M* = 94.02, *SD* = 4.27) and those who performed blocked sequences (*M* = 95.59, *SD* = 2.51; *U* = 992.50, *p* = .229). Those who were tested on interleaved sequences either received blocked or interleaved training the day before, however training condition did not impact accuracy on Day 2 (*M*_*II*_ = 93.34, *SD*_*II*_ = 4.44; *M*_*BI*_ = 94.74, *SD*_*BI*_ = 4.06; *U* = 280.5, *p* = .234). Similarly, training condition did not impact accuracy on Day 2 for those who were tested on blocked sequences (*M*_*BB*_ = 95.67, *SD*_*BB*_ = 2.32; *M*_*IB*_ = 95.52, *SD*_*IB*_ = 2.72; *U* = 197.50, *p* = .968).

Participants who received blocked practice explicitly recalled on average more sequential items per sequence (*M* = 4.18, *SD* = 2.48) than those who had received interleaved practice (*M* = 3.17, *SD* = 2.13); *t*(81) = -1.996, *p* = .049). Though 84.3% of participants reported noticing a pattern, only 30.1% of participants correctly noticed there were three sequences. Before categorizing participants into implicit or explicit learners, we conducted a two-way ANCOVA to control for recall score. We found a significant main effect of training condition (*F*(1,78) = 38.06, *p* < .001), a significant main effect of testing condition (*F*(1,78) = 10.895, *p* = .001), and a significant interaction after controlling for recall score (*F*(1,78) = 11.565, *p* = .001). The covariate was not significantly related to performance, indicating that a participants’ knowledge about the sequence had no significant impact on performance or the benefit of interleaved practice (*F*(1,78) = 3.02, *p* = .08). Since our original interest was purely implicit motor sequence learning, we then separated groups based on a cutoff score denoting chance performance. Learners were sorted into two groups based on their explicit recall score: implicit (*n*=40) and explicit (*n*=43). See Table 1 for breakdown of individual group *n*s.

**Table 1:** Number of participants per condition and learner type for Experiment 1

Condition	Implicit Learners	Explicit Learners	Total
II	14	8	22
BB	7	12	19
IB	9	12	21
BI	10	11	21
Total	40	43	83

*Notes.* Since Implicit/Explicit sorting was determined post hoc, there are uneven numbers in each group. On average, Implicit Learners recalled an average of 1.64 items per sequence (*SD* = 1.32) while Explicit Learners recalled an average of 5.54 items per sequence (*SD* = 1.28).

*II* Interleaved/Interleaved, *BB* Blocked/Blocked, *IB* Interleaved/Blocked, *BI* Blocked/Interleaved

**Fig. 2:**
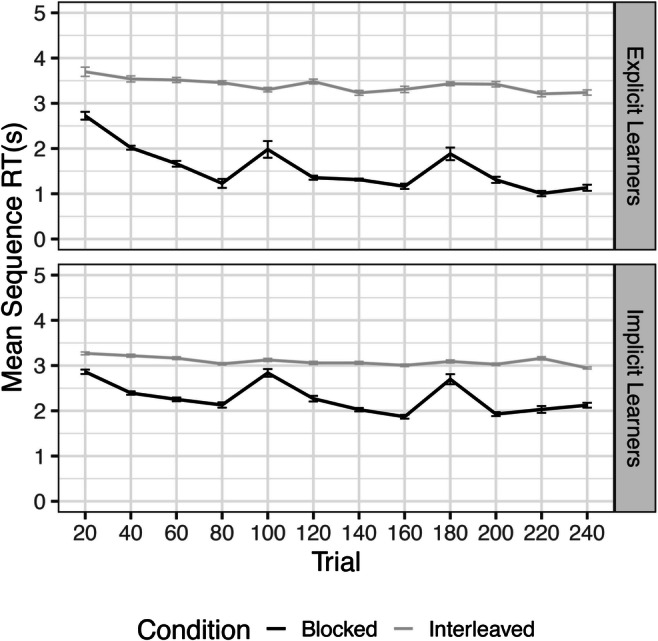
Learning curves during training for Explicit and Implicit Learners, Experiment 1. Significant decreasing trends in all groups reveal learning over 240 trials

For explicit learners, we found significant decreasing monotonic trends for both the interleaved training group (*τ* = -.242, *p* < .0001) as well as the blocked training group (*τ* = -.442, *p* < .0001) (Fig. 2). Thus, both groups showed improvement on the SRTT during training on Day 1. This was also true for implicit learners, who also showed significant decreasing monotonic trends for both the interleaved training group (*τ* = -.272, *p* < .0001) as well as the blocked training group (*τ* = -.336, *p* = .001) (Fig. 2).

Additionally, we conducted one-sample t-tests to determine if learning difference scores differed significantly from zero, which would indicate a change in performance from the beginning to the end of training on Day 1. Participants in the blocked training condition showed faster RTs at the end of training as compared with the beginning of training (*M* = -.9585, *SD* = .7576); *t*(39) = -8.00, *p*<.001, *d* = -1.27. However, using this measure, there was only a weak trend for a significant difference in RT between the first and last sequences in the interleaved condition (*M* = -.199, *SD* = 0.78). *t*(42) = -1.6657, *p* = 0.10, *d* = -.25, despite the significantly decreasing monotonic trends in RT across the session in the interleaved practice condition for both the implicit and explicit learners. To compare learning difference scores between practice conditions, an independent-samples t-test was conducted. As expected, we found that the blocked training group (*M* = -.9585, *SD* = .7576) did show faster learning during training compared with the interleaved group (*M* = -.199, *SD* = 0.78); *t*(81) = 4.478, *p* < .001, *d* = .98. This is consistent with findings in the CI literature in which blocking stimuli in the absence of high contextual interference facilitates fast performance improvements.

**Fig. 3:**
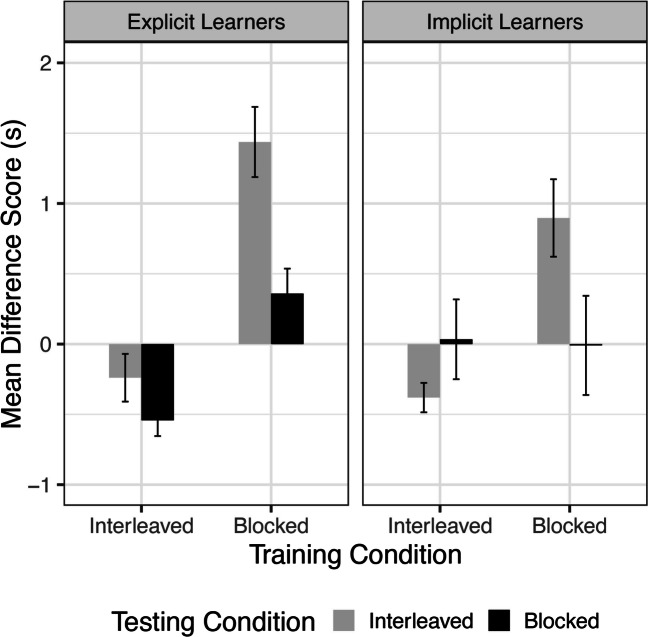
Mean difference score per condition. Positive difference scores represent increased reaction times (RTs) on Day 2 as compared to Day 1, indicating worse performance. Error bars represent ± SEM

A three-way ANOVA was conducted that examined the effect of training condition, testing condition, and learner type (explicit, implicit) on mean RT difference scores. There was a significant main effect of training condition on difference scores, (*F*(1, 75) = 39.539 , *p* < .001, η^2^ = 0.274), with less forgetting from Day 1 to Day 2 for participants who had received interleaved training (Fig. 3). Participants who trained in the interleaved condition had a negative difference score, indicating improved performance (*M* = -0.313, *SD* = 0.554). Participants who trained in the blocked condition instead showed a positive difference score, indicating poorer performance from Day 1 to Day 2 (*M* = 0.726, *SD* = 0.938). There was also a significant main effect of testing condition, (*F*(1, 75) = 9.538, *p* = .003, η^2^ = 0.066), with greater forgetting for participants who received interleaved testing on Day 2. Participants who received interleaved testing had a mean positive difference score (*M* = 0.408, *SD* = 1.017), while participants who were tested with blocked sequences had a negative mean difference score (*M* = -0.049, *SD* = 0.748).

These main effects were qualified by two statistically significant interactions. First, we observed an interaction between the effects of training condition and testing condition on difference scores, *F*(1,75) = 11.948, *p* < .001, η^2^ = 0.083. An analysis of simple effects showed that testing condition did not significantly affect difference scores when participants were trained in the interleaved condition (*F*(1) = 0.027, *p* = .870). However, testing condition did significantly affect the difference scores when participants were trained in the blocked condition (*F*(1) = 20.056, *p* < .001). Participants who trained with interleaved sequences were able to retain or improve performance regardless of testing condition, while participants who trained with blocked sequences did worse when tested with interleaved sequences.

Additionally, we observed an interaction between the effects of training condition and explicit knowledge on difference scores, *F*(1, 75) = 4.915, *p* = .03, η^2^ = 0.034. Simple effects analysis showed that learner type did affect difference scores when subjects trained with blocked sequences (*F*(1) = 4.529, *p* = .037), but not when subjects trained with interleaved sequences (*F*(1) = 1.082, *p* = .302). Explicit learners in the blocked training condition had higher difference scores (i.e., more forgetting) than implicit learners, suggesting that explicit learning of the sequences might hinder performance on the delayed test, especially when the sequences were first practiced in a blocked fashion.

In implicit learners, the BI and II groups showed difference scores significantly different than zero. The BI group showed increased RTs on Day 2, indicating forgetting, while the II group showed decreased RTs on Day 2, indicating some consolidation of learning ((*t*(9) = 3.255, *p* = .009; *t*(13) = -3.633, *p* = .003, respectively). The groups tested with blocked sequences on Day 2 (BB and IB) showed similar RTs across the delay, suggesting good retention of learning across training conditions when sequences were tested in blocks, with no differences between the end of training and the beginning of the test (*t*(6) = -.0265, *p* = .979; *t*(8) = .119, *p* = .910, respectively).

In explicit learners, only the BI and IB groups showed difference scores significantly different than zero, in a positive and negative direction, respectively. The BI group showed increased RTs on Day 2 (*t*(10) = 5.757, *p* = .0001), while the IB group demonstrated decreased RTs on Day 2, which reflect faster performance for blocked sequences (*t*(11) = -4.849, *p* = .0005). Difference scores of participants in the II and BB groups did not significantly differ from zero, suggesting good retention from Day 1 (*t*(7) = -1.412, *p* = .201; *t*(11) = 2.026, *p* = .068, respectively).

Hours of sleep were assessed; however, participants reported adequate numbers, with little variation (*M*= 6.86 , *SD* = 1.19), and so it was excluded as a covariate.

### Discussion

In this experiment, we used the SRTT to examine the effect of interleaved practice on implicit learning of fine motor sequences after a 1-day delay. We hypothesized that interleaved practice would result in poorer initial performance but superior long-term retention, in line with the CI effect (Shea & Morgan, 1979). Consistent with the past findings, we found participants who practiced blocked sequences were much faster than those who practiced interleaved sequences, but they were left unprepared for interleaved sequences at test, especially those with explicit sequence knowledge (Magill & Hall, 1990; Shea & Morgan, 1979; Wright et al., 2016).

We found that interleaved practice may reduce interference from explicit knowledge. An interaction between training condition and learner type (explicit or implicit) revealed that retention was similar for explicit and implicit learners when they practiced interleaved sequences. But for those who practiced blocked sequences, explicit learners were especially impacted, suggesting that explicit knowledge of the sequences may hinder SRTT performance only when sequences are presented in a blocked fashion. This finding is consistent with previous work demonstrating that explicit sequence knowledge can be detrimental to speeded performance in a visuomotor task (Tanaka & Watanabe, 2017) as well as on the SRTT (Reber & Squire, 1998; but see Willingham et al., 2002). It may be that interleaving sequences ameliorates the possible interference that can arise from explicit knowledge, perhaps because practice with high CI encourages general, non-sequence-specific learning that is more immune to intrusions of explicitly learned sequence elements.

The CI effect in motor learning has been reliably demonstrated with explicit sequence learning, but there had been little evidence to suggest that interleaving could benefit sequence learning that occurred without awareness. We found similar results in implicit and explicit learners, in that the CI effect was most pronounced when interleaved sequences were used in the retention test. Even with little to no awareness of any structure within the key presses, those who practiced interleaved sequences showed consolidation of learning when tested with interleaved sequences on Day 2. Those who practiced with blocked sequences were slower when those same sequences were interleaved on Day 2, indicating “forgetting” of sequences that participants were not aware they had learned. These results seem to be in line with the Specificity of Learning hypothesis in that for implicit learners, a consistent practice and testing condition (II) led to better learning than an inconsistent one (BI) (Barnett et al., 1973). But those in the IB and BB groups both showed retention, rather than forgetting and consolidation, respectively. This suggests that, similar to other motor studies, our results are more in line with the Variability of Practice hypothesis that emphasizes the role of task variation (e.g., through interleaving) in supporting the learner’s ability to abstract a generalizable schema they can apply to other skills or tasks (Schmidt, 1975; Schmidt et al., 1990; Shea & Kohl, 1990). Blocked practice might result in less flexible learning that is specific to practice conditions, even without conscious awareness of sequences or structure in the task.

In Experiment 1, interleaved practice led to more flexible retention of practiced sequences in that there was no forgetting for sequences that were presented in either a blocked or interleaved order. In Experiment 2, we examined whether interleaved practice leads to greater generalization of sequence learning to performance of new sequences. Positive transfer to novel tasks or contexts is a crucial goal in many training situations as one often cannot train on every possible task variation or in every possible context.

## Experiment 2

### Method

#### Participants

125 right-handed young adults were enrolled in the study (96 female; age 18–30 years, *M* = 20.6, *SD* = 1.8). Participants were UCLA undergraduate students and were given course credit for their participation. All participants gave informed consent using an institutionally approved consent form. Participants were excluded if they had a history of neurological or psychiatric disease or if they were taking neuroactive medication that could affect sensory processing, movement, or cognition. All participants reported that they were right-handed, but degree of handedness was not assessed. Participants were excluded if they had an accuracy lower than 80% on either day (Willingham et al., 2000). A total of 30 participants were excluded for low accuracy (*n* = 16), computer error (*n* = 4), and failing to complete the experiment (*n* = 10). Our final subject pool consisted of 95 right-handed young adults (72 female, age 18–30 years, *M* = 20.5, *SD* = 1.7).

#### Study design

The study design and task is largely the same as Experiment 1, except that the three sequences presented on Day 2 were novel (e.g., DDD...EEE...FFF for blocked testing or DFEFEDFED….for interleaved testing; Fig. 1). Subjects were randomly assigned to one of four training/testing conditions (BB, BI, IB, II). Explicit knowledge of sequences was assessed as in Experiment 1, except that subjects were asked if they remembered sequences from either Day 1 or Day 2.

#### Data analysis

Sequence RTs were calculated by summing the eight key press RTs for correct sequences. Data analysis was largely similar to Experiment 1, except for how the difference score was calculated. To assess transfer, we compared the summed RTs of the first ten presentations of each sequence in the blocked condition or the first 30 sequences in the interleaved condition, and subtracted these from the first ten presentations of each new sequence in the blocked test condition, or the first 30 sequences in the interleaved test condition. Positive transfer would be indicated by faster RTs at the beginning of Day 2 compared to initial performance on Day 1, while negative transfer, or interference, would be indicated by slower RTs. Similar RTs for the beginning of Day 1 and Day 2 would reflect a lack of transfer. We utilized an independent t-test to assess mean items recalled per sequence in both training groups in the explicit knowledge test. We assessed learning during Day 1 training and Day 2 testing using the Mann-Kendall test, a nonparametric test for monotonic trends. Learning difference scores were also calculated by subtracting the RT of the first thirty trials of Day 1 from the RT of the last thirty trials from Day 1.

We also conducted a 2 × 2 between-subjects ANOVA to assess the effect of training and testing conditions on transfer difference scores (beginning of Day 2 – beginning of Day 1). In addition, we conducted an ANCOVA with recall score as the covariate. We also conducted one-sample t-tests to assess which groups (BB, BI, IB, II) had transfer scores that significantly differed from zero.

### Results

On Day 1, a Mann-Whitney test indicated that participants who practiced interleaved sequences were significantly less accurate (*M* = 91.07, *SD* = 5.13) than participants who practiced blocked sequences (*M* = 94.71, *SD* = 3.45; *U* = 1660.00, *p* < .001). This was also found to be true on Day 2 (*M*_*I*_ = 94.21, *SD*_*I*_ = 3.36; *M*_*B*_ = 95.72, *SD*_*B*_ = 3.07; *U* = 1455.50, *p* = .014). Those who were tested on interleaved sequences either received blocked or interleaved training the day before, however training condition did not impact accuracy on Day 2 (*M*_*II*_ = 94.00, *SD*_*II*_ = 3.51; *M*_*BI*_ = 94.44, *SD*_*BI*_ = 3.23; *t*(48) = 0.47, *p* = .642). Similarly, training condition did not impact accuracy on Day 2 for those who were tested on blocked sequences (*M*_*BB*_ = 96.30, *SD*_*BB*_ = 2.51; *M*_*IB*_ = 94.98, *SD*_*IB*_ = 3.59; *U* = 308.50, *p* = .185).

Explicit recall of sequences was low for both training groups, with no subjects producing more than an average of three sequential items per sequence. On average, subjects recalled fewer than two sequential items per sequence, which is essentially chance recall of sequence elements, indicating that sequence knowledge was substantially implicit. Unlike in Experiment 1, no participants were excluded from analysis based on substantial explicit sequence knowledge. Though 75.7% of participants reported that they noticed a pattern, only one participant correctly recalled there were six sequences in the entire experiment. There was no significant difference in item recall between participants who trained with interleaved sequences (*M* = 1.41 , *SD* = 1.18) and those who trained with blocked ones (*M* = 1.85 , *SD* = 1.13) ; (*t*(96) = -1.8504 , *p* = .067, *d* = -0.38). Both groups recalled fewer than two sequential items per sequence on average, demonstrating that all participants learned the sequences implicitly. The reduced amount of explicit sequence knowledge shown by the participants compared to Experiment 1 is likely because Experiment 2 involved six sequences per participant (three sequences on Day 1 followed by three novel sequences on Day 2) and one, not two, sessions of practice on each set of sequences.

**Fig. 4:**
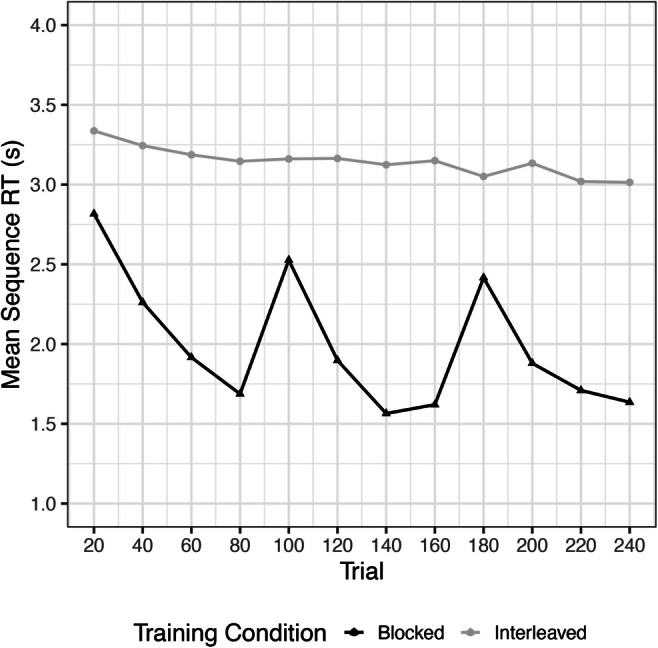
Learning curves during training, Experiment 2. Significant decreasing trends in both groups reveal learning over 240 trials

**Fig. 5:**
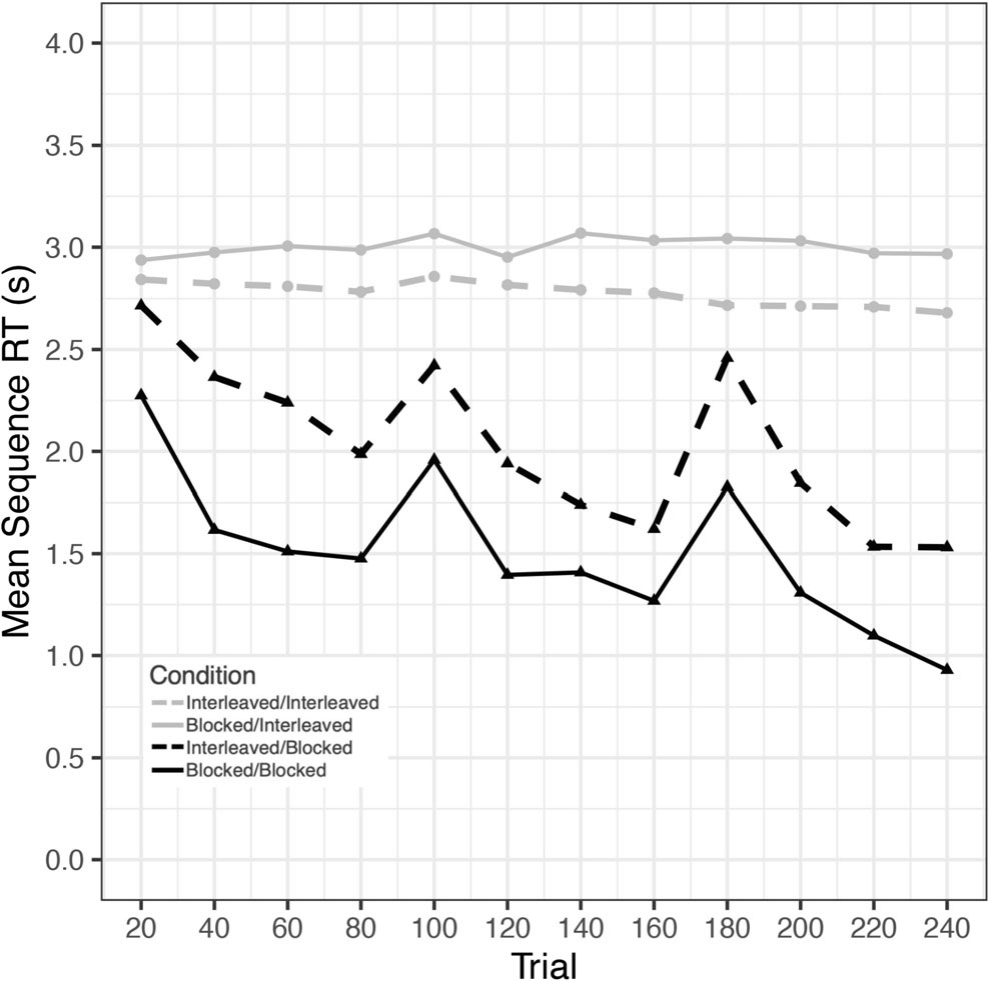
Learning curves over Day 2, Experiment 2. Significant decreasing monotonic trends were found in all groups, except for Blocked-Interleaved, suggesting that blocked training may hinder learning of novel interleaved sequences. Each point is an average of 20 trials

We found significant decreasing monotonic trends in RT for both the interleaved training group (*τ* = -.431, *p* < .0001) as well as the blocked training group (*τ* = -.39, *p* < .0001) (Fig. 4). This indicates that both groups showed learning of the sequences over Day 1. On Day 2, significant decreasing monotonic trends were found in all groups, except for BI, indicating that blocked training may hinder new learning of interleaved sequences (Table 2; Fig. 5).

**Table 2:** Results of Mann-Kendall trend test for testing day, Experiment 2

Condition	Kendall’s Tau	P-value
II	-.283	<.0001
BB	-.539	<.0001
IB	-.531	<.0001
BI	.0595	.17

*II* Interleaved/Interleaved, *BB* Blocked/Blocked, *IB* Interleaved/Blocked, *BI* Blocked/Interleaved

We conducted one-sample t-tests to determine if difference scores from the beginning to the end of training differed significantly from zero, which would indicate a significant change in performance from the beginning to the end of training. Similar to our findings in Experiment 1, participants in the blocked training condition showed faster RTs at the end of training as compared with the beginning (*M* = -1.19; *SD* = .71); *t*(48) = -11.74, *p* < .001; *d* = -1.68. However, unlike our previous findings, we found that participants in the interleaved training condition did show a significant decrease in RT, demonstrating successful learning as evidenced by a negative difference score (*M* = -.31; *SD* = .64); *t*(45) = -3.2573, *p* = .0021, *d* = -.48. Consistent with our findings in Experiment 1, we found that the blocked training group (*M* = -1.19; *SD* = .71) showed a greater decrease in RT during training compared to the interleaved group (*M* = -.31; *SD* = .64); *t*(93) = 6.31, *p* < .001, *d* = 1.30.

**Fig. 6:**
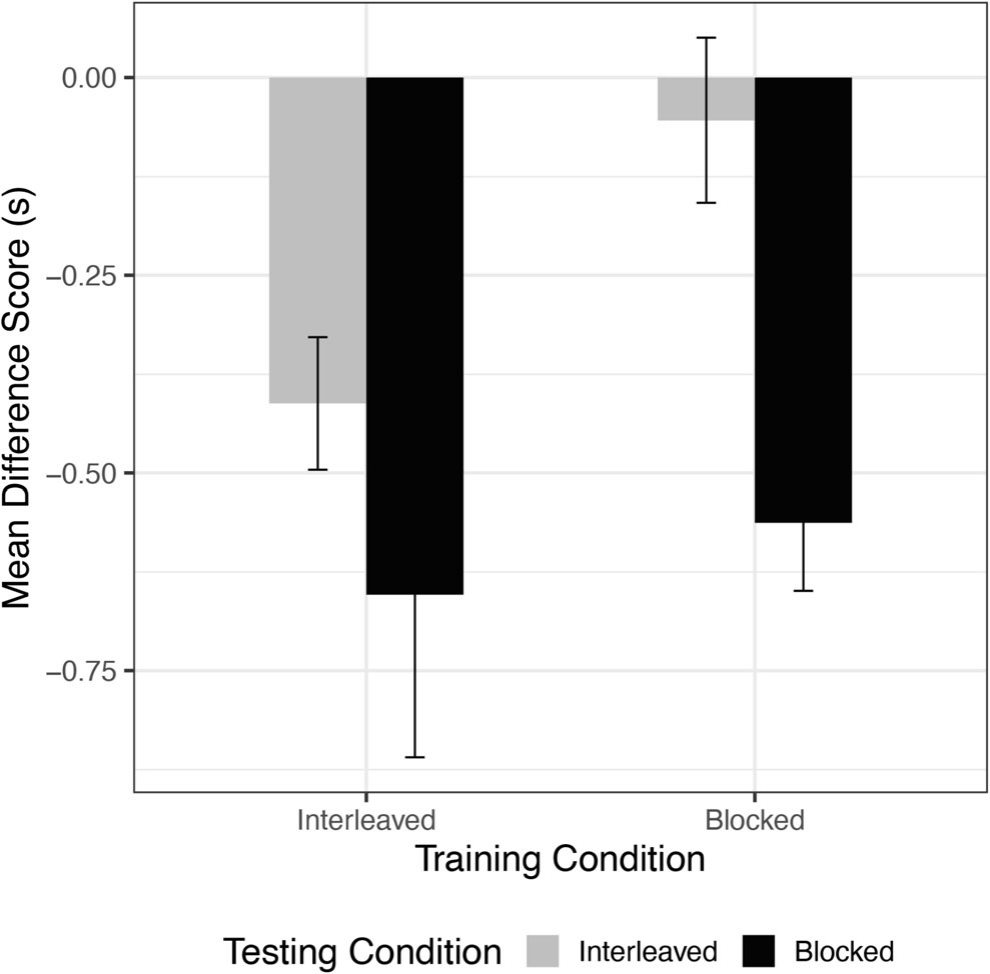
Transfer learning difference score per condition. Note: Negative difference scores indicate faster reaction times (RTs) on Day 2 as compared to Day 1. All groups except for Blocked/Interleaved demonstrated positive transfer to novel sequences. Error bars represent ± SEM

We next examined transfer learning with a two-way ANOVA of Training condition (Interleaved, Blocked) and Testing condition (Interleaved, Blocked). We found a significant main effect of testing condition, *F*(1,91) = 9.689, *p* = .003,η^2^= .092 (Fig. 6). Subjects tested with new sequences in the blocked condition showed greater transfer to the new sequences, as evidenced by a negative difference score (*M* = -.608, *SD* = .588). Subjects tested in the interleaved condition also had a negative difference score, but of a smaller magnitude (*M* = -.233, *SD* = .585). There was a trend for a main effect of training condition (*F*(1,91) = 3.469, *p* = .066,η^2^= .033), with participants receiving interleaved training exhibiting numerically greater transfer to new sequences. The interaction between training and testing conditions was not significant (*F*(1,91)=1.225, *p*=.27,η^2^= .012). However, post hoc Tukey tests showed significant differences between BI and IB groups (*M* = 0.599, *p* = .005) as well as between BI and BB groups (*M* = 0.509, *p* = .015). Those in the BI group showed significantly worse transfer performance than those in the IB and BB groups. This indicates that for blocked practice alone, the type of testing condition affects transfer performance. An ANCOVA controlling for recall score reveals a significant main effect of testing condition (*F*(1,90) = 8.895, *p* = .0004,η^2^ = .090). Again, the covariate was not found to be significantly related to performance (*F*(1,90) = .057, *p* = .811,η^2^ = .0006).

One-sample t-tests revealed that all groups, except for BI, had transfer scores that differed significantly from zero (*M*_*BI*_ = -.054, *SD*_*BI*_ =.511,*t*(23) = -.516 , *p* =.611). Thus, II, IB, and BB all showed positive transfer to novel sequences (*M*_*II*_ = -.412, *SD*_*II*_ = .428, *M*_*IB*_ = -.654, *SD*_*IB*_ = .919, *M*_*BB*_ = -.563, *SD*_*BB*_ = .431; *p*s <.01). In other words, participants in the blocked training condition showed transfer to the new sequences only when they were presented in a blocked order. Participants who received interleaved training showed significantly faster RTs for the new sequences at the beginning of Day 2 compared with the beginning of Day 1 regardless of test condition.

Like the previous experiment, hours of sleep were assessed but were again excluded as a covariate due to sufficient hours of sleep and little variation (*M* = 7.07 , *SD* = 1.49).

### Discussion

In Experiment 2, we observed implicit sequence learning and transfer for both blocked and interleaved practice conditions in the SRTT. After interleaved practice, there was substantial transfer to performance of new sequences for both testing conditions. After blocked practice, significant positive transfer occurred if new sequences were blocked, but not if they were presented in an interleaved order. This mirrors our results in Experiment 1 in that blocked practice seemed to be vulnerable to testing condition, while interleaved practice prepared participants for both testing conditions. These results suggest that blocked practice of sequences results in implicit learning that is relatively specific to the mode of practice.

While blocked practice did result in positive transfer to new blocked sequences, these participants performed new interleaved sequences at a similar level to their performance at the onset of initial practice. In contrast, interleaved practice of sequences resulted in implicit learning that facilitated performance of new sequences presented in either a blocked or interleaved fashion. In this way, interleaved practice resulted in learning that was general to the SRTT rather than encapsulated in the practiced sequences, similar to previous findings (Müssgens & Ullén, 2015). Additionally, prior research using sequential rule paradigms has found that extensive training with a cognitive task was associated with more errors in a transfer task in which the same rules were used but in a different order, suggesting that sequential expectations about a task can interfere with transfer performance (Woltz et al., 2000). Blocked practice may thus be more susceptible to violated sequential expectations, resulting in poorer transfer learning, especially when sequences are interleaved.

This result extends recent findings that prior interleaved practice can result in broader learning benefits that are not specific to the practice session. Interleaved practice is usually associated with poorer acquisition and superior retention, however experience with prior interleaved practice may actually improve acquisition of a novel skill (Hodges et al., 2014; Kim et al., 2016, 2018). Individuals who underwent prior interleaved practice showed faster acquisition of a novel task as compared with those with prior blocked practice, thereby mitigating the costs normally associated with high CI during learning. This suggests that experience with high CI may facilitate the rate of learning beyond an isolated practice session, perhaps because the learner is able to apply strategies gleaned from interleaved practice to novel motor tasks. It is possible that prior interleaved practice enabled individuals to generate many different motor programs that could aid future learning of similar motor skills (Kim et al., 2018). This may also be reflected in our finding that all groups showed learning during Day 2, except those in the BI group. When novel sequences were interleaved at test, prior blocked practice seemed to hinder participants from learning, while those with prior interleaved practice demonstrated successful learning of novel sequences in both testing conditions.

## Summary and concluding discussion

We investigated two hypotheses about the effects of interleaved practice on implicit sequence learning. First, we tested whether interleaved practice of sequences leads to greater retention than blocked practice by examining the effect of practice schedule on sequence RT tested the following day after practice. Next, we tested whether interleaved practice of sequences lead to greater positive transfer to novel sequences than blocked practice by examining the effect of practice schedule on performance of novel sequences the day after practice. We found support for the benefit of interleaved practice on both retention and transfer of implicit sequence learning, indicating that the benefit of interleaved practice does not depend on explicit memory retrieval, but also holds for implicit fine motor learning over a delay. Explicit knowledge of the sequence was detrimental to retention when the sequences were blocked, but not when they were interleaved, suggesting that contextual interference may protect against the interference of explicit knowledge on performance.

The SRTT was used here as it is a relatively simple task that has been used extensively to study implicit learning (Robertson, 2007). We used eight-item sequences as these are less likely to be learned explicitly than shorter sequences (Meissner et al., 2016; Song et al., 2008). Nevertheless, in Experiment 1, where participants practiced the same three sequences for two days, many participants gained at least some awareness of the sequence, particularly in the BB condition. Because more participants became aware of some elements of the sequences in the blocked conditions, it is possible that the unaware participants in the blocked condition differed in some other systematic way from those who gained awareness. It is unclear why some individuals gained partial explicit knowledge of blocked sequences while others did not – perhaps the large number of practice trials (80 per sequence) were repetitive enough for some observant individuals to notice structure in the task (Willingham, 2001). The largest group of implicit learners (n=14) was found in the Interleaved/Interleaved condition (Table 1). Our results seem consistent with the idea that high CI can encourage implicit learning due to the increased working memory load from frequent task-switching that may make explicit learning more difficult (Rendell et al., 2011). In tasks in which explicit knowledge could hinder performance, such as the SRTT (Reber & Squire, 1998), CI may facilitate performance by inducing learners to rely on implicit knowledge. Similar results were found by Rendell et al. (2011), who examined participants’ performance of two gross motor skills of different difficulties while completing a secondary task. Interestingly, participants who practiced a kicking skill with high contextual interference performed exceptionally well under dual-task conditions, suggesting greater implicit learning. The secondary task may require participants to rely on a lesser amount of attentional control and thus these performance gains are due to implicit, rather than explicit, learning. Interleaving tasks may make it more difficult to acquire explicit knowledge, and thus the learner may learn implicitly during acquisition, which in turn maybe more effective for retention and transfer performance. Notably, this result only applied to the more complex skill of kicking, so task difficulty is an important consideration when examining CI (Albaret & Thon, 1998; Farrow & Buszard, 2017; Guadagnoli & Lee, 2004). The Challenge Point Framework predicts that the strength of the CI effect is partially determined by task difficulty; namely that it is more robust with low difficulty tasks (Guadagnoli & Lee, 2004). Since Rendell et al. (2011) only observed the CI effect in the more challenging task, these results seem inconsistent with this framework. In the present study, we used a low difficulty task and were able to observe benefits of CI during practice. Future research should aim to clarify these disparate findings and specifically manipulate task difficulty under varying levels of contextual interference. It is possible that with more complex sequences there would be little or no benefit of CI.

Recent research indicates that frequent error processing in addition to task switching increases cognitive effort and may encourage implicit learning (Broadbent et al., 2017). In both experiments presented here we found that those in the interleaved condition were less accurate than those in the blocked condition on Day 1, which may lend some credence to the theory that frequent error processing may occur with high CI (Broadbent et al., 2017). However, on Day 2 of Experiment 1, accuracy did not differ between the two conditions, and we did not observe the costs normally associated with interleaved practice, consistent with past research (Hodges et al., 2014) which may reflect a general learning benefit of interleaving that results in improved skill acquisition (Kim et al., 2016, 2018).

In Experiment 2, we focused on transfer to new sequences. This type of transfer may be conceptually related to playing a new piece of music after extensive practice of a different piece. Implicit learning has been thought to be inflexible and not amenable to transfer (Dienes & Berry, 1997; Sanchez et al., 2015), though our results suggest that positive transfer of implicit motor sequences may be facilitated by introducing high CI during acquisition. The present results are the first to show a benefit for interleaving in the positive transfer of learned sequences in which the lack of awareness of learning was assessed, indicating that explicit processes may not be required to observe this effect. Past research has found that explicit knowledge can reduce errors but increase RTs, hindering transfer performance overall (Benson et al., 2011; Tanaka & Watanabe, 2017). However, error processing may be an important component of the learning process as errors allow learners to compare between the actual versus expected outcomes of response selection, which can then inform hypotheses and rules that learners may generate to improve performance (Maxwell et al., 2001; Rabbitt, 1966). Thus, fewer errors due to explicit knowledge may actually hinder successful long term retention despite superior acquisition performance. As discussed above, this is in line with the idea that interleaving increases working memory load, presumably by task switching and error processing, which encourages an implicit form of learning (Broadbent et al., 2017; Rendell et al., 2011).

Though promising, our results should be cautiously interpreted due to some limitations. Sample size for both experiments was analogous to similar studies, but the substantial number of explicit learners in Experiment 1 led to a smaller sample size for the implicit learner group. However, we felt that dichotomizing was essential as we were interested in whether the CI effect persists in the absence of explicit knowledge, as implicit learning is an important component of long-term skill retention in both healthy adults (Howard et al., 2004; Mazzoni & Krakauer, 2006) and for clinical populations with explicit memory deficits (Curran, 1997; Gabrieli et al., 1993) or those with chronic stroke (Boyd & Winstein, 2004; Dimyan & Cohen, 2011; Wadden et al., 2017). Though prior literature has used similar methods to exclude those with explicit awareness, we acknowledge that this categorization does not address the question of how the effects of interleaving are impacted by increasing levels of awareness of learning. Additionally, some participants who were classified as explicit learners may have only gained explicit knowledge of the sequences towards the end of the retention test, and thus may have learned the sequences implicitly during Day 1. Future research could examine the effects of CI when learning is more clearly implicit in all participants, such as learning with a concurrent task (Grafton et al., 1995; Nejati et al., 2008) or a more complex probabilistic sequence (Du et al., 2016; Song et al., 2007).

Fatigue was a potential factor that impacted performance given the large number of trials during training and testing. Extended practice may cause fatigue and participants might fail to show learning despite successful skill acquisition (Eysenck, 1965). Performance might improve if fatigue could be mitigated with intervals of rest, but previous work demonstrated that adding breaks to the SRTT did little to change performance in implicit learners (Robertson et al., 2004). Additional evidence suggests measuring motor skill learning via RT difference scores is unaffected by fatigue (Heuer et al., 1998). However, it is possible that fatigue impacted participants’ motivation or effort, given the number of participants who were excluded based on low accuracy or failing to complete the experiment, despite the SRTT’s relative simplicity. Future research may utilize fewer trials, as implicit sequence transfer may occur even with a shorter learning period (Tanaka & Watanabe, 2015).

In sum, the present results add to the literature showing the benefits of interleaved practice on learning and transfer and extend them by providing evidence that this effect is also observed in implicit fine motor sequence learning. Blocked practice of sequences in real world skills, such as repetitively playing single pieces when learning an instrument, may appear to be effective in that performance will improve within the practice session. However, this type of practice may not be as effective for mastering the instrument as interleaved practice of different pieces. Furthermore, these results suggest that patients with deficits in explicit memory can still benefit from high CI to successfully learn and retain novel motor skills.

### Open practices statement

The datasets generated during and/or analyzed during the current study are available from the corresponding author on reasonable request. Neither of the experiments were preregistered.

## Declarations

### Author note

This research was supported in part by the National Science Foundation (BCS- 1634157 to B.K.) and UCLA’s Graduate Research Mentorship Fellowship (to J.S.).
